# A pancreatic ductal adenocarcinoma subpopulation is sensitive to FK866, an inhibitor of NAMPT

**DOI:** 10.18632/oncotarget.10776

**Published:** 2016-07-22

**Authors:** Marine Barraud, Jonathan Garnier, Celine Loncle, Odile Gayet, Charlotte Lequeue, Sophie Vasseur, Benjamin Bian, Pauline Duconseil, Marine Gilabert, Martin Bigonnet, Aurélie Maignan, Vincent Moutardier, Stephane Garcia, Olivier Turrini, Jean-Robert Delpero, Marc Giovannini, Philippe Grandval, Mohamed Gasmi, Mehdi Ouaissi, Veronique Secq, Flora Poizat, Nicolas Guibert, Juan Iovanna, Nelson Dusetti

**Affiliations:** ^1^ Centre de Recherche en Cancérologie de Marseille (CRCM), INSERM U1068, CNRS UMR 7258, Aix-Marseille Université and Institut Paoli-Calmettes, Parc Scientifique et Technologique de Luminy, Marseille, France; ^2^ Hôpital Nord, Marseille, France; ^3^ Institut Paoli-Calmettes, Marseille, France; ^4^ Hôpital de la Timone, Marseille, France; ^5^ CIC1409, AP-HM - Nord University Hospital, Aix-Marseille University, Marseille, France; ^6^ Hospices Civils de Lyon, Lyon, France

**Keywords:** pancreatic cancer, NAMPT, FK866, chemotherapy

## Abstract

Treating pancreatic cancer is extremely challenging due to multiple factors, including chemoresistance and poor disease prognosis. Chemoresistance can be explained by: the presence of a dense stromal barrier leading to a lower vascularized condition, therefore limiting drug delivery; the huge intra-tumoral heterogeneity; and the status of epithelial-to-mesenchymal transition. These factors are highly variable between patients making it difficult to predict responses to chemotherapy. Nicotinamide phosphoribosyl transferase (NAMPT) is the main enzyme responsible for recycling cytosolic NAD^+^ in hypoxic conditions. FK866 is a noncompetitive specific inhibitor of NAMPT, which has proven anti-tumoral effects, although a clinical advantage has still not been demonstrated. Here, we tested the effect of FK866 on pancreatic cancer-derived primary cell cultures (PCCs), both alone and in combination with three different drugs typically used against this cancer: gemcitabine, 5-Fluorouracil (5FU) and oxaliplatin. The aims of this study were to evaluate the benefit of drug combinations, define groups of sensitivity, and identify a potential biomarker for predicting treatment sensitivity. We performed cell viability tests in the presence of either FK866 alone or in combination with the drugs above-mentioned. We confirmed both inter- and intra-tumoral heterogeneity. Interestingly, only the *in vitro* effect of gemcitabine was influenced by the addition of FK866. We also found that NAMPT mRNA expression levels can predict the sensitivity of cells to FK866. Overall, our results suggest that patients with tumors sensitive to FK866 can be identified using NAMPT mRNA levels as a biomarker and could therefore benefit from a co-treatment of gemcitabine plus FK866.

## INTRODUCTION

Treating patients with pancreatic ductal adenocarcinoma (PDAC) is a challenge due to the poor prognosis of this disease and its resistance to chemotherapies. This can be partially attributed to a dense stroma barrier surrounding the tumor and the lack of vessels, which decrease drug delivery to the tumor site, also limiting access to oxygen and nutrients [1]. PDACs also present epithelial-to-mesenchymal transition and the presence of cancer stem cells is a sign of aggressiveness and chemoresistance [2]. Chemoresistance is particularly difficult to overcome due to genetic heterogeneity both between pancreatic tumors, and within each tumor. These factors can render patients completely unresponsive to a given chemotherapy and in those cases that do respond the selection of resistant clones within the tumor can cause cancer relapse. Hypoxic conditions and limited access to nutrients induce cancerous cells to adapt their metabolism to survive and grow in this hostile environment [3]. One of the mechanisms activated is the Warburg's effect, characterized by increased glycolysis and lactate overproduction. This process, is also known as aerobic glycolysis and promotes tumor growth [4]. The high rates of aerobic glycolysis perturb NAD^+^ metabolism, altering the NADH/NAD^+^ redox ratio. Indeed, instead of eventually transferring electrons from NADH to the mitochondrial respiratory chain, a large proportion of cytosolic NAD^+^ is regenerated by reducing pyruvate to lactate. NAD^+^ is an essential coenzyme involved in numerous metabolic pathways and energy transduction of nearly every cell. It mediates signaling events and participates in the regulation of many biological processes, including transcription, cell cycle progression, apoptosis, DNA repair and metabolic regulation [5]. Cellular NAD^+^ levels have been recognized as a homeostatic process with immediate effects on cellular metabolism. A stable level is maintained by a finely regulated balance between NAD^+^ degradation, NAD^+^ synthesis and NAD^+^ recycled through a salvage pathway. Pancreatic cancer cells rely heavily on the NAD^+^ salvage pathway for their metabolism. Two enzymes which play major roles in this pathway are nicotinamide phosphoribosyl transferase (NAMPT) and nicotinamide mononucleotide adenylyl transferase (NMNAT1) [6, 7]. NAMPT is the most active enzyme catalyzing the rate-limiting step of nicotinamide condensation with 5-phosphoribosyl-1-pyrophosphate to yield nicotinamide mononucleotide, then NMNAT1, leading to NAD^+^ from nicotinamide mononucleotide and ATP. NAMPT is up-regulated in a number of solid tumors and is involved in angiogenesis by inducing vascular endothelial growth factor [8, 9].

FK866 is a noncompetitive highly specific inhibitor of NAMPT and clinically interesting as it is a potent antitumor drug both *in vitro* and *in vivo* [10]. Many recent studies provide evidence that it selectively inhibits growth of various kinds of cancer cells, with no effect on normal cells [8]. It causes cellular death by apoptosis [11] and induces autophagy. Its effects on autophagy are potentiated by chloroquine and antagonized by 3-methyladenine or by down-regulating autophagy-related proteins [12, 13]. Interestingly, NAMPT inhibition sensitizes pancreatic adenocarcinoma cells to tumor-selective, PAR-independent metabolic catastrophe and cell death induced by β-lapachone [14] and FK866 led to either tumor regression or stabilization in a preclinical trial [11]. However, in five clinical trials testing three specific NAMPT inhibitors (FK866, CHS828 and GMZ1777), no significant tumor remission was observed in a total of 104 patients. Clinical studies on FK866 have revealed that due to its short half-life in circulation, prolonged treatment regimens are required, inducing toxicity to proliferating hematopoietic cells. Therefore, the efficiency of NAD^+^ depleting drugs, such as NAMPT inhibitors, when used alone are expected to be low due to insufficient tumor-selectivity [15-17]. For this reason, FK866 has also been tested as an additive drug to other well-known chemotherapies. It increased the chemosensitivity of gastric cancer cells to 5-Fluorouracil (5FU) [18], potentiated the effects of cisplatin and etoposide in neuroblastoma cell lines [13] and massively reduced the overall metabolic activity in xenografts, impairing PDAC growth [19].

Combining drugs to treat chemoresistant cancers, such as PDAC, subsequently became a promising alternative. The best example in PDAC was the advent of Folfirinox (5FU, oxaliplatin and irinotecan) that produced the longest improvement in survival ever seen in a phase III clinical trial of patients with advanced pancreatic cancer [20, 21]. This drug combination led to increased cell death, a reduction of drug resistance, while allowing the use of lower doses and therefore fewer side effects. Here, we sought to increase the efficiency of killing pancreatic cancer cells via an association of drugs which target two different cellular processes (i.e.: metabolism and genomic stability). We investigated the effect of the NAMPT inhibitor, FK866, on PDAC-derived primary cell cultures (PCCs) and determined whether it can potentiate the effects of three frequently used chemotherapeutical agents: gemcitabine, 5FU and oxaliplatin. We also attempted to determine groups of sensitivity to these drug combinations and identify a screening biomarker (companion test) capable of predicting this sensitivity.

## RESULTS

### FK866 sensitivity is heterogeneous in PCCs from PDAC patients

**Figure 1:**
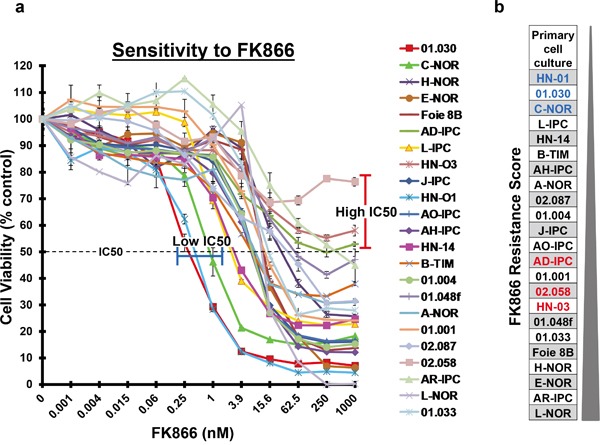
FK866 sensitivity of 23 PDAC-derived PCCs **a.** Twenty-three PDAC-derived PCCs were treated for 72 h with increasing concentrations of FK866 ranging from 0 to 1000 nM. The horizontal dotted line indicates 50% cell viability. PCCs with the highest sensitivity are highlighted by a blue line and those with an IC50>1000 nM by a red line. **b.** PCCs were scored on their resistance to a fixed FK866 concentration of 3.9 nM, from the most sensitive to the most resistant. PCCs in blue are the ones that present the lowest IC50 and those in red have an IC50>1000 nM.

Twenty-three PDAC patients were included in this study and patient's distribution representing a normal PDAC cohort is indicated in Table 1. An increase in the number of operated patients was observed due to the fact that xenografts from surgical specimens grow better than biopsies. PCCs were obtained from patient-derived xenografts in nude mice. These cells were submitted to increasing concentrations of FK866 (from 0 to 1000 nM) to determine their sensitivity by plotting dose-response curves. We established a chemogram (i.e.: determination of the cell viability as a function of the chemotherapeutic drug concentration) for each patient-derived PCC. Using this approach we were able to estimate their relative chemosensitivity by comparing their half maximal inhibitory concentrations (IC50s). Notably, the three PCCs most sensitive to FK866 were: HN-01 (IC50 = 0.41 nM), 01.030 (IC50 = 0.30 nM) and C-NOR (IC50 = 0.85 nM), whereas 02.058, HN-03 and AD-IPC were the three most resistant, all with IC50s > 1000 nM (Figure 1a). This manner of analyzing data could not determine scores for those PCCs that did not achieve an IC50, even at very high FK866 concentrations. We observed maximal variability in the sensitivity of PCCs to FK866 at a concentration of 3.9 nM. We next decided to score the PCCs sensitivity based on cell viability following treatment with 3.9 nM FK866 (Figure 1b). HN-01, 01.030 and C-NOR remained the most sensitive PCCs, however the most resistant were: L-NOR, AR-IPC and E-NOR. We used this scoring method for the next experiments in this study. Overall, our data reveals that each PDAC-derived PCC has its own sensitivity to FK866 with a huge range of IC50s (from 0.30 to >1000 nM), suggesting a very high response variability among patients. Importantly, the dose-response curves depict a cell viability plateau for almost every PCC, even with high doses of FK866, indicating intra-tumoral heterogeneity in terms of sensitivity to the FK866 treatment (Figure 1). As sensitivity to FK866 could be associated with PCC replication time, this was calculated and analyzed. Doubling times varied between 16.46±5.33h for L-IPC and 44.63±6.07h for 02.087p (Supplementary Figure 1). All PCCs actively replicate during the time of chemosensitivity determination and no correlation was observed between doubling time and FK866 sensitivity and between doubling time and NAMPT expression level (data not shown). We also analyzed clinical characteristics of FK866 sensitive and resistant patients and we did not find any correlation.

**Table 1:** Patients Distribution: Clinical characteristics for the 23 patients included in the study

Patients Distribution		
Patients		23
Sex	Male	16 (70%)
	Female	7 (30%)
Age	Mean	65
	Median (Min-Max)	66 (42-87)
Personal History of Cancer	No	18 (79%)
	Yes	5 (21%)
Surgery		12 (52%)
Unresectable		11 (48%)
Tumor Location	Head	14 (61%)
	Neck	1 (4%)
	Body	0
	Tail	8 (35%)
Locally Advanced		3 (13%)
Metastasis	Liver	4 (17%)
	Lung	2 (8.5%)
	Carcinomatosis	2 (8.5%)
Tumor Status at Diagnosis	Stage I	0
	Stage IIA	2 (8.5%)
	Stage IIB	9 (40%)
	Stage III	4 (17%)
	Stage IV	8 (34.5%)
Overall Survival (Months)	Median (Min-Max)	12.1 (1.2-38.8)

### FK866 increases sensitivity to gemcitabine, but not 5FU or oxaliplatin, in a group of PCCs from PDAC patients

**Figure 2:**
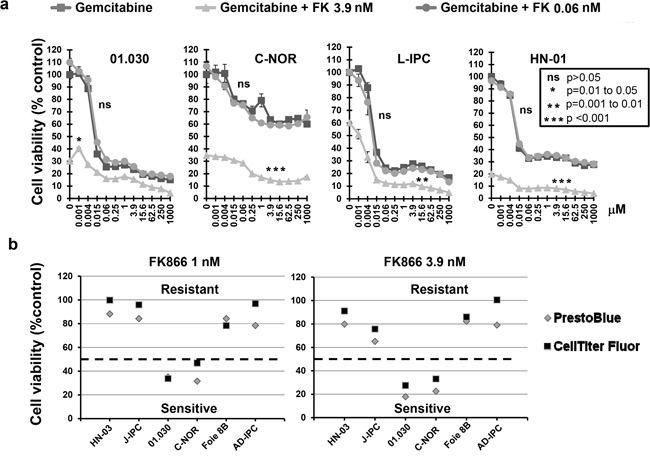
a. Effect of FK866-gemcitabine co-treatment on four PCCs most sensitive to FK866 Dose response curves represent cell viability with increasing concentrations of either gemcitabine alone or in combination with two concentrations of FK866 (0.06 and 3.9 nM). Three independent experiments were done (each one in triplicate) showing similar results. Significant difference between curves (p≤0.05) was analyzed by the Mann-whitne test. **b.** Comparison of cell viability measured with either PrestoBlue™ or Cell Titer-Fluor™ after FK866 treatment. Cell viability was measured with either PrestoBlue™ or Cell Titer-Fluor™ after 3 days treatment with two concentrations of FK866 (1 and 3.9 nM). The dotted horizontal line indicates 50% cell viability.

**Figure 3:**
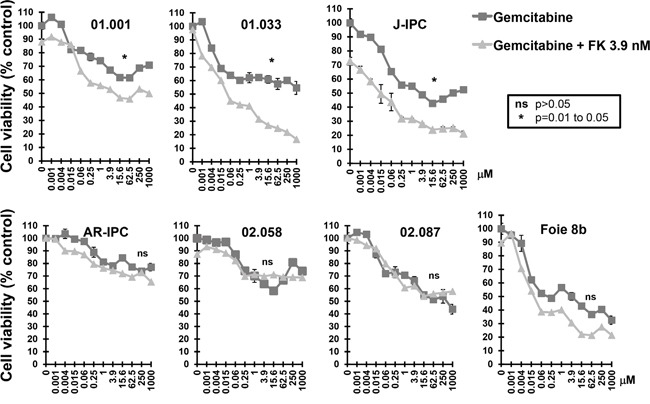
Sensitivity of FK866 resistant PCCs to co treatment with FK866-gemcitabine Dose response curves represent cell viability with increasing concentrations of gemcitabine either alone or in combination with two concentrations of FK866 (3.9 nM). Three independent experiments were done (each one in triplicate) showing similar results. Significant differences between curves (p≤0.05) were analyzed by the Mann-whitne test.

In order to study FK866 in combination with other drugs, we tested two different FK866 concentrations (0.06 and 3.9 nM) with a series of 11 increasing concentrations of three genotoxic drugs (5FU, oxaliplatin and gemcitabine). We selected a concentration of 0.06 nM FK866 as this was the maximal dose that caused a minimal effect in all cells tested. Conversely, the dose of 3.9 nM caused the most variable effect between the 23 PCCs (see Figure 1). We decided to continue the study using the 4 most sensitive PCCs to FK866 (01.030, C-NOR, L-IPC and HN-01). A combination of 0.06 nM FK866 with different gemcitabine concentrations had no significant effect on cell viability (Figure 2a). However, 3.9 nM FK866 significantly decreased cell viability in all 4 sensitive PCCs tested. We therefore concluded that treatment with 3.9 nM FK866 can enhance the effect of gemcitabine, which was not observed at the lower concentration (0.06 nM). The PrestoBlue method we used to measure cell viability is based on mitochondrial activity (see Material and Methods). As FK866 could influence mitochondrial activity, potentially inducing misinterpretation of data, we decided to compare results obtained with PrestoBlue™ with another method that measures living cells by their protease activity (CellTiter-Fluor™). As shown in Figure 2b, the 6 PCCs tested (HN-03, J-IPC, 01.030, C-NOR, Foie 8B and AD-IPC) using two concentrations of FK866 (1 and 3.9 nM), confirmed that results obtained with PrestoBlue™ and CellTiter-Fluor™ are not significantly different. Therefore all measurements of cell viability were subsequently carried out using the PrestoBlue™ method. We next analyzed the effect of combining FK866 with gemcitabine on 7 PCCs that show different degrees of resistance to FK866 (01.001, 01.033, J-IPC, Foie 8B, AR-IPC, 02.058 and 02.087) and observed an important heterogeneity (Figure 3). For PCCs 01.001, 01.033 and J-IPC, there was a clear synergistic effect of combining the drugs, which was not apparent for the PCCs AR-IPC, 02.058, 02.087 and Foie 8B. Most of the PCCs tested (8 out of 11 PCCs) were more sensitive to this co-treatment.

**Figure 4:**
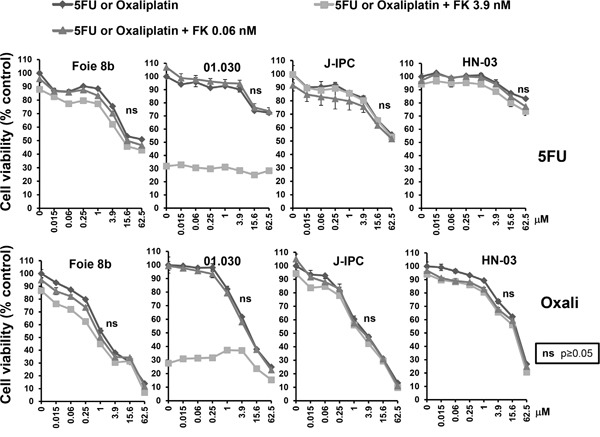
Sensitivity of PCCs to co-treatment with FK866-5FU and FK866-oxaliplatin Dose response curves represent cell viability at increasing concentrations of 5FU or oxaliplatin, either alone or in combination with two concentrations of FK866 (0.06 and 3.9 nM). Three independent experiments were done (each one in triplicate) showing similar results. Significant differences between curves (p≤0.05) were analyzed by the Mann-whitne test.

In another set of experiments, 5FU and oxaliplatin were both combined with FK866 at either 0.06 or 3.9 nM. Figure 4 shows results obtained using the PCCs Foie 8B, 01.030, J-IPC and HN-03. Curiously, a combination of NAMPT inhibitor with either 5FU or oxaliplatin resulted in no significant effect, with the exception of 01.030, although only at high concentrations of oxaliplatin (15.6 and 62.5 nM). Interestingly, neither 5FU nor oxaliplatin are clinically used as a monotherapy since they have proven to be almost non-effective, contrary to gemcitabine. Therefore, addition of FK866 could be more advantageous in combination with gemcitabine treatment instead of 5FU or oxaliplatin.

### NAMPT transcriptional level predicts FK866 sensitivity

**Figure 5:**
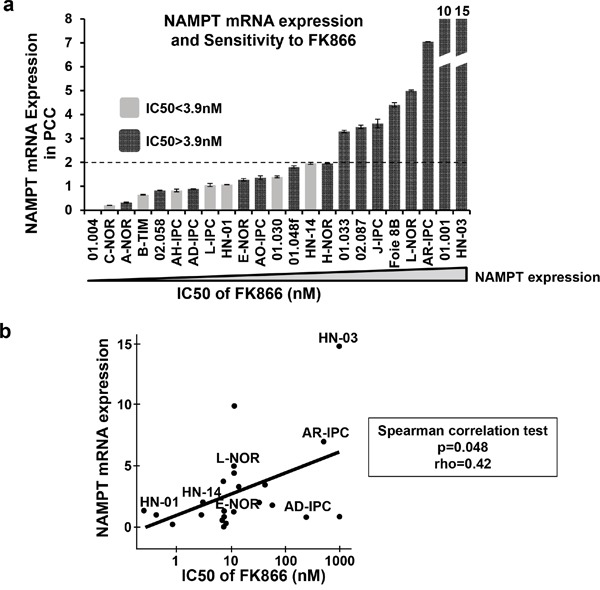
NAMPT mRNA expression levels in PCCs **a.** PCCs were ordered according to their NAMPT expression level as determined by RT-qPCR (the lowest NAMPT expression level is on the left; highest on the right). The dark grey bars are FK866 resistant PCCs (FK866 IC50>3.9 nM); light grey bars are FK866 sensitive PCCs (FK866 IC50<3.9 nM). **b.** Correlation between FK866 IC50 and NAMPT mRNA expression for the 23 PCCs with Spearman correlation test (p=0.048); rho=0.42.

Next, we hypothesized that NAMPT, as a specific target of FK866, could predict drug sensitivity. We therefore quantified NAMPT at the transcriptional level in PCCs by quantitative real-time PCR (RT-qPCR). We plotted the mRNA expression level in arbitrary units from 0 to 15 (Figure 5a) and used IC50 values to reflect and compare the global sensitivity of PCCs. As shown in Figure 5a, all PCCs expressing over 2 arbitrary units of NAMPT presented IC50s > 3.9nM. On the contrary, only 53% of PCCs (8 out of 15) showed IC50s > 3.9 nM with an NAMPT expression level of less than 2 arbitrary units. Therefore, our results indicate that resistance to FK866 positively correlates with the expression level of NAMPT transcript. In Figure 5b we plotted results obtained by a regression analysis between NAMPT mRNA expression and FK866 IC50 in PCCs. We observed a positive correlation, indicating that PCCs which express higher levels of NAMPT have an increased resistance to FK866 treatment. These results strongly suggest that the primary culture of pancreatic epithelial cells does not significantly modify NAMPT transcriptional expression. In some cases we observed PCCs (especially E-NOR or AD-IPC) with low NAMPT mRNA expression and high resistance to FK866, we have no clear explanation for that but we observed that in all cases highly NAMPT expressing cells have IC50>3.9. We also analyzed the putative association between patient survival Vs NAMPT expression and Vs FK866 sensitivity and we did not find any correlation. We analyzed other clinical characteristics in particular differentiation status of xenograft and we did not find any association.

### Metabolic status of PDAC-derived cells does not correlate with NAMPT expression level

**Figure 6:**
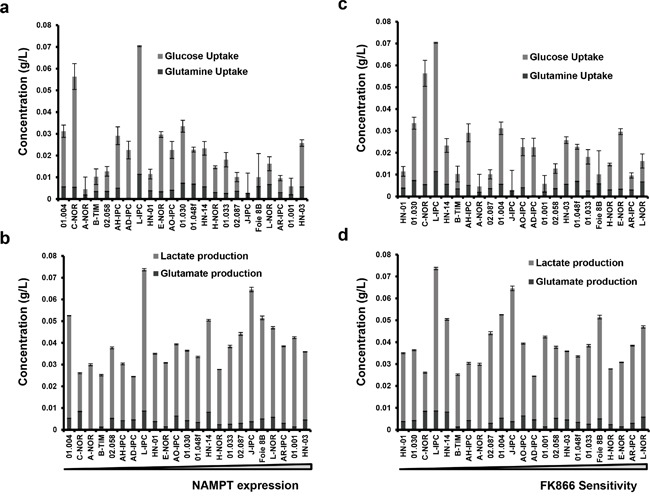
Correlation between NAMPT expression or FK866 sensitivity with either glucose/glutamine uptake or lactate/glutamate production Primary cell cultures were ordered according to either their NAMPT transcriptional level **a** and **b.** or their FK866 IC50 **c** and **d.** For each of them we measured glucose and glutamine accumulated uptake, as well as lactate and glutamate production. The values obtained were normalized by the amount of protein.

NAD^+^ is a coenzyme which plays an essential role in metabolism within tumoral cells. Its synthesis is accomplished by a salvage pathway implicating NAMPT. Since NAMPT expression level and sensitivity to FK866 correlate, we hypothesized that tumoral cells with a high metabolism, i.e consuming or producing more metabolites with higher levels of NAMPT, would die at a faster rate than metabolically neutral cells. We therefore analyzed the relationships between either NAMPT expression or FK866 sensitivity with glucose/glutamine consumption and lactate/glutamate production for the 23 PCCs (Figure 6). We found no correlation between these metabolic parameters and FK866 sensitivity. Contrary to our hypothesis, high metabolism does not predict either FK866 sensitivity or NAMPT expression.

### Higher sensitivity of PDAC-derived cells to co-treatment (gemcitabine + FK866) compared to gemcitabine alone

**Figure 7:**
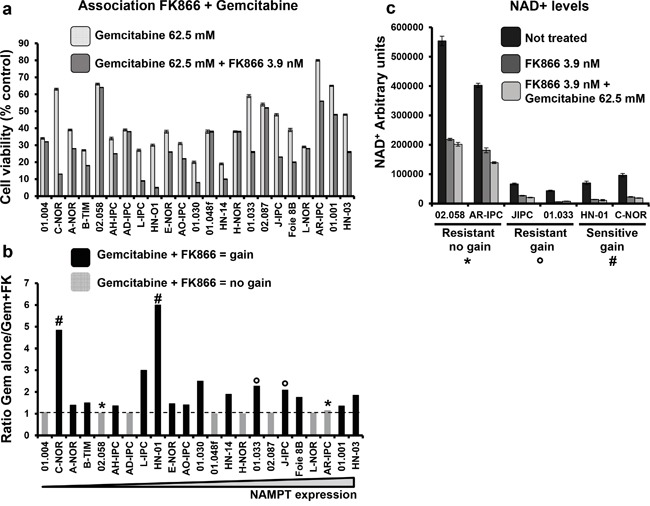
Co-treatment of FK866-gemcitabine **a.** Cell viability was measured and indicated as percentage of the control (untreated) after 3 days treatment with either 62.5 mM gemcitabine or 62.5 mM gemcitabine + 3.9 nM FK866. **b.** Ratio of treatment with gemcitabine alone / gemcitabine +FK866. The transversal line indicates ratio = 1. Values > 1 indicate a gain with the combined treatment and are represented by black bars on the graph. No gain from co-treatment is indicated by the light grey bars. **c.** NAD^+^ levels measured in: FK866 resistant cells presenting gain (*) or not (°) after treatment with gemcitabine; and FK866 sensitive cells (#). Cells were treated or not with FK866 alone or with FK866 + gemcitabine and NAD^+^ levels measured 24h later. Values are presented as arbitrary units and were normalized to cell number (see Material and Methods). Experiments were repeated 3 times and performed in triplicate.

We next studied the sensitivity of PCCs to treatments with gemcitabine alone versus gemcitabine plus FK866 in all 23 PCCs. Gemcitabine was used at a concentration of 62.5 μM, demonstrated to be efficient in the chemograms, and FK866 at 3.9 nM (Figure 7a). As shown in Figure 7b, the combined treatment (gemcitabine + FK866) synergistically decreased the cell viability of 16 PCCs from 23 (69.6%), each with a ratio of > 1 (cell viability after treatment with gemcitabine alone / cell viability after combined gemcitabine + FK866). However, we observed no correlation between this benefit of combined treatment and NAMPT expression levels. We then analyzed the effect of FK866 alone or combined with gemcitabine on the intracellular levels of NAD^+^ (see Material and Methods). As shown in Figure 7C, 02.058 and AR-IPC cells which are resistant to FK866 and do not show any decrease in their viability after gemcitabine + FK866 treatment present high NAD^+^ levels in all conditions (treated or not with FK866 or with FK866 + gemcitabine). Differently, cells resistant to FK866 and with a decrease in their viability after combined treatment FK866+gemcitabine (J-IPC, 01.033) as well as FK866 sensitive cells (HN-01 and C-NOR) show low levels of NAD^+^ in all conditions. We finally examined clinical characteristics of patients that could take advantage of a combined treatment against the others and we did not find any common characteristic between them. Overall, these results suggest that most PDAC patients could take advantage of co-treatment with gemcitabine + FK866. In addition, quantification of NAMPT mRNA expression could be used as a potential biomarker for determining the sensitivity of tumors to FK866, despite NAMPT levels not predicting a benefit of a combined treatment of gemcitabine-FK866. Interestingly high NAD^+^ levels seem to designate cells that will not present a decrease in cell viability with the FK866-gemcitabine combined treatment.

## DISCUSSION

The main aims of this study were to define whether there are differences in sensitivity of PDACs to a metabolic drug either alone or in combination; and if so, to determine a way to identify the most sensitive patients by characterizing tumors at the molecular level. The results presented in this paper have revealed a group of tumors that appear more sensitive to FK866, a specific inhibitor of NAMPT. An interesting aspect of this work is the efficient strategy we employed to include 23 consecutive patients with PDAC tumors in the study by collecting samples from both surgical specimens and EUS-FNA biopsies and preserving them as xenografts in immunosuppressed mice. We studied NAMPT mRNA expression profiles of these patient-derived PCCs using RT-qPCR and found a correlation between NAMPT expression and sensitivity to FK866. Finally, and most importantly, we discovered that FK866 improves the effect of gemcitabine *in vitro*, although not 5FU or oxaliplatin.

NAMPT is a fascinating target for the chemotherapeutic treatment of PDAC for a number of reasons Firstly, the recycling of NAD^+^ is vital for cancer cells due to a high need of ADP ribosylation to repair DNA, preserve genome stability, and maintain telomeres [22]. This is why NAD^+^ dependence renders cancer cells more sensitive to NAMPT inhibition than normal cells. Secondly, NAMPT allows NAD^+^ synthesis by a salvage pathway in a dose dependent manner, and NAMPT is known to be increased in several cancers [23]. Finally, NAD^+^ synthesis is increased by DNA damage in primary culture cells [24].

FK866 is an interesting compound since its mechanism of action is well established and several clinical trials have been performed in patients with different cancers, including several solid tumors. Previous studies indicate that metabolic reprogramming is not only the consequence of cancer cells proliferation and growth; it is a direct function of oncogenesis and consequently an oncogenesis motor [25, 26]. Cancer cells are also NAD-dependent as NAD^+^ is an important cofactor in the oxidative phosphorylation chain as well as a substrate for enzymes involved in genomic stability, apoptosis, cell signaling, stress tolerance and metabolism. NAMPT is the main enzyme responsible for maintaining NAD^+^ levels in tumors and may be the “Achilles' heel” to study for future chemotherapy.

An important point to note from our study is that the IC50 for FK866 in patient-derived PCCs varied greatly from 0.295 nM to >1000 nM. A group of patients expressing high levels of NAMPT mRNA were relatively resistant to the treatment with FK866 suggesting that its expression was not completely inhibited by FK866, perhaps for a stoichiometric reason. Accordingly, we found that PCCs expressing intermediary or lower levels of NAMPT mRNA were frequently associated with higher levels of sensitivity to the treatment with FK866. We hypothesize that this group with higher sensitivity is dependent on NAMPT levels and the amount expressed by the cell can be inhibited by FK866. Altogether, our data suggest that levels of NAMPT in human tumors can be used to predict sensitivity to FK866 treatment. Consistent with this, it has been previously suggested that NAMPT expression could play a role in the response to FK866 [5, 19]. For example, on fibrosarcoma cells NAMPT overexpression has been shown to protect cells against FK866 treatment [27]. Furthermore, NAMPT inhibition using a siRNA in the presence of GMX1778, another NAMPT inhibitor, increased apoptosis of different cancer cells. In addition, NAMPT expression showed an inverse correlation to GMX1778 toxicity [28].

It is unlikely that NAMPT inhibition would be solely used as a therapeutic approach for treating patients with a PDAC since FK866 at high concentrations is very toxic, as anticipated from its mechanism of action which affects basic functions of both cancerous and normal cells. Therefore, the only possibility is to use FK866 in combination with cytotoxic drugs to potentiate their effect. Accordingly, the second point to be noted from this work is the significant potentiation of gemcitabine on cell viability by co-treatment with FK866, yet surprisingly, this added benefit was almost negligible when combined with 5FU or oxaliplatin. This was unexpected since previous studies have shown a potentiation of the effects of 5FU by FK866 on gastric cancer cells and of cisplatin by FK866 on neuroblastoma cells [13, 18]. These results suggest that the enhancement of the effects of cytotoxic drugs by FK866 is most likely dependent on cancer cell type. Moreover intracellular NAD^+^ depletion after cell treatment with FK866 induces autophagy [12, 29]. VMP1-Mediated Autophagy is also induced by gemcitabine promoting apoptosis [30]. Both effects could be additive increasing cell death and explaining the synergistic effect of FK866 and gemcitabine. Another putative explanation could be that gemcitabine induces the DNA damage response and in consequence PARP-1 activity [31]. PARP-1 needs NAD^+^ as a substrate for auto poly-ADP-ribosylation (PARylation) and activation. PARPs are highly responsive proteins; their enzymatic activity can increase up to 500-fold after DNA consuming up to 80–90% of the intracellular NAD^+^ stores [32]. As NAD^+^ in depleted by FK866 treatment, PARP-1 could become inactive and thus increase the effect of gemcitabine on pancreatic cancer cells. On another hand differently to gemcitabine, autophagy induction protects cancer cells against oxaliplatin and 5FU-induced cell death [33, 34]. This could explain the absence of synergistic effect combining Oxaliplatin and 5FU with FK866 on pancreatic cancer primary cultures.

FK866 has been shown to exert strong effects on cancer cell metabolism, such as glycolysis, pentose phosphate pathway, tricarboxylic acid cycle, serine biosynthesis and attenuating glycolysis at the glyceraldehyde 3-phosphate dehydrogenase step due to reduced availability of NAD^+^ for the enzyme [35]. We therefore hypothesized that FK866 could be more efficient in tumors with increased glycolysis or glutaminolysis. However, contrary to this we were unable to demonstrate a correlation between either glucose/glutamine consumption or lactate/glutamate production and sensitivity levels of PCCs to FK866 treatment. This suggests that pancreatic cancer cell metabolism is not mandatory to the sensitivity to FK866 treatment. Nevertheless some particular points are worth discussing, in particular the one observed for the L-IPC tumor that presents the highest lactate production. This patient was diagnosed with an anaplastic carcinoma of the pancreas, one of the most aggressive forms of pancreatic cancer and his survival time after diagnosis was of only 5.28 month. On the other hand J-IPC presents one of the highest survival times in this cohort (25.74 months). These two cases represent interesting but only isolated observations and more of these uncommon patients would be necessary to include in the study in order to draw solid conclusions about pancreatic cancer aggressiveness and metabolism.

Finally, we tested the efficiency of NAMPT in depleting NAD^+^ (Figure 7c). Cells presenting higher basal levels of NAD^+^, also presented elevated levels of NAD^+^ after FK866 and FK866 + gemcitabine treatments. Interestingly FK866 sensitive and resistant cells showing a decreased viability after gemcitabine treatment also showed lower levels of NAD^+^ before and after the different treatments, suggesting that basal levels of NAD^+^ in pancreatic tumoral cells could predict if the patient will take advantage of a combined FK866 and gemcitabine treatement. Tumoral cells with high NAD^+^ basal levels should be resistant to FK866 and did not show any decrease in viability with a combination FK866 + gemcitabine in contrast to FK866 alone.

In addition, our results present a clinical interest since RT-qPCR for measuring NAMPT mRNA expression can be routinely performed either on systematic echo-endoscopy-guided biopsies at diagnosis or on specimens after surgery. A simple and reproducible test to predict the sensitivity of a tumor to FK866 would greatly aid clinicians in their daily decisions of treatment. With lower doses of combined drugs, patients would be partly spared from side effects while receiving an appropriate and personalized treatment. Patients needing gemcitabine to treat a metastatic or an operated PDAC could therefore benefit from a co-treatment of FK866-gemcitabine.

In conclusion, our data demonstrate that: the IC50 of FK866 to kill PCCs varied more than 4000 fold between sensitive and resistant cells; sensitivity to FK866 is dependent on an optimal cellular level of NAMPT mRNA expression which could be exploited as a companion test to select suitable patients for treatment; and finally, FK866 potentiates the effect of gemcitabine on PDAC-derived PCCs.

## MATERIALS AND METHODS

### PDAC samples and cell culture

Consent forms of informed patients were collected and registered in a central database. The tumor tissues used for xenograft development were deemed excess to that required for the patient's diagnosis. Two types of samples were obtained: endoscopic ultrasound-guided fine-needle aspiration (EUS-FNA) biopsies from patients with unresectable tumors; and tumor tissues from patients undergoing surgery. A total of 23 patients were included, all without treatment before biopsy or surgery. PDAC samples were mixed with 100 μl of matrigel (BD Biosciences) and implanted with a trocar (10 Gauge, Innovative Research of America, Sarasota, FL) into the subcutaneous right upper flank of an anesthetized and disinfected mouse. When tumors reached 1 cm^3^, mice were sacrificed and tumors removed.

To obtain primary cell cultures of these tumors, xenografts were split into several small pieces and processed in a biosafety chamber: after a fine mincing, they were treated with collagenase type V (ref C9263; Sigma) and trypsin/EDTA (ref 25200-056; Gibco, Life Technologies) and suspended in DMEM supplemented with 1% w/w penicillin/streptomycin (Gibco, Life Technologies) and 10% fetal bovine serum (Lonza). After centrifugation, cells were re-suspended in serum free ductal media (SFDM) adapted from Schreiber *et al*. [36] without antibiotic and incubated at 37°C in a 5% CO_2_ incubator. Primary cultured cells were splitted many times until obtaining 4x10^7^ cells (representing 20 vials with 2x10^6^ cells each). This was considered passage zero. At this stage only human cancerous epithelial cells were present in the culture. Cells were then splitted a maximum of 6 more times to avoid other alterations associated with passages. Mouse contaminant cells do not replicate any more after a few passages.

### Chemograms

Cells were screened for their chemosensitivity to FK866 (Sigma-Aldrich, France). They were grown in SFDM without nicotinamide for 72 h. Five thousand cells per well were plated in 96-well plates in SFDM medium without nicotinamide. Twenty-four hours later the media was supplemented with increasing concentrations of FK866 and incubated for an additional 72 h period. Each experiment was done in triplicate and repeated at least two times. These cells were treated for 72 h with increasing concentrations of FK866 ranging from 0 to 1000 nM (in a progression by a factor4). FK866 was next tested at a given concentration in association with increasing concentrations of gemcitabine, oxaliplatin or 5FU (0 to 1000 μM).

### Viability assays

Cell viability was estimated after addition of PrestoBlue™ reagent (Life Technologies) for 3 h, following the PrestoBlue™ cell viability reagent protocol provided by the supplier. Live-cell protease activity was also measured using CellTiter-fluor™ (Promega, France). For both techniques cell viability was measured by fluorescence. FK866 sensitivity was close for each primary cell culture if cell viability was estimated with either PrestoBlue™ or CellTiter-Fluor™. PrestoBlue™ monitors mitochondrial function alteration as an indicator of cellular necrosis and apoptosis. Cells with an active metabolism reduce resazurin to resorufin due to NADPH, FADH and NADH electrons. It causes a color change detected by fluorometry. Following the strategy published in 2013, cell viability was measured using CellTiter-Fluor™ as an alternative and unrelated method [37]. Since similar results were observed from the two methods, data was obtained in this paper using PrestoBlue™.

### Metabolite consumption and production

Glucose and glutamine uptake and lactate production by cells were quantified in the cell medium after 48 h of incubation. Glutamate production was quantified in cell lysate 48 h after medium change. Measurements were carried out with the enzymatic membrane system bioanalyser robot YSI 2950 (Biochemistery Analyser, Life Sciences). Results were normalized to protein amount.

### NAD+ measurement

NAD+ was measured using the NAD^+^/NADH™ bioluminescent Assay (Promega). Cells were chosen according to their response to FK866 and FK866+Gemcitabine treatments. 3 groups: 1-cells resistant to FK866 that do not present any differences in viability when treated additionally with gemcitabine (02.058; AR-IPC); 2-Cells that are resistant to FK866 and that present a decrease in viability when treated with gemcitabine (J-IPC; 01.033) and 3-cells that are sensitive to FK866 and that show a decreased viability when treated with gemcitabine (HN-01; C-NOR) see Figures 2 and 3. Five thousand cells were seeded in each well of 96 well-plates, culture medium used was Nicotinamide-free SFDM. Twenty-four hours after plating, culture media was supplemented with 3.9nM FK866 or a combination of 3.9nM FK866 and 62.5μM Gemcitabine. NAD^+^ was measured twenty-four hours later. NAD+ values were normalized according to viable cell number after treatment.

### RT-qPCR

One μg RNA from xenografts was reverse transcribed using the Go Script reagent (Promega) according to manufacturer's instructions. RT-qPCR for NAMPT mRNA was performed in a Stratagene cycler using Takara reagents. Primer sequences were specific to the gene NAMPT (forward 5'-TTCGGTTCTGGTGGAGGTTT-3', reverse 5'-AGACGTTAATCCCAAGGCCA-3').

### Statistical analysis

Data are expressed as means ± SD from at least two independent experiments. Data were analyzed using an unpaired *t* test. Significance was set at p<0.05.

The manuscript has been revised for the English by an independent scientific language editing service.

## SUPPLEMENTARY MATERIALS FIGURE


