# Supplementary material for: A pancreatic ductal adenocarcinoma subpopulation is sensitive to FK866, an inhibitor of NAMPT
Source: Oncotarget. 2016 Jul 22;7(33):53783–96. doi: 10.18632/oncotarget.10776 (PMC5288221; doi:10.18632/oncotarget.10776)
Supplement: Supplementary file 1 [file oncotarget-07-53783-s001.pdf]

A pancreatic ductal adenocarcinoma subpopulation is sensitive to FK866, an inhibitor of NAMPT

SUPPLEMENTARY FIGURE

Doubling Times for PCC

Patient	Doubling time	Standard Deviation
L-IPC	16.46	5.33
01.033	17.75	5.7
Foie-8B	20.53	3.84
01.001	26.18	3.34
01.004	26.34	6.61
AH-IPC	27.12	5.13
AD-IPC	27.59	8.88
B-Tim	28.05	1.64
C-NOR	28.53	6.29
J-IPC	29.39	4.32
HN-03	29.51	0.68
E-NOR	29.69	10.82
L-NOR	30.34	4.09
H-NOR	31.37	1.84
A-NOR	33.03	5.79
AO-IPC	33.33	2.58
01.030	33.54	3.19
HN-14	34.39	10.42
HN-01	40.3	2.86
01.048f	41.94	7.08
AR-IPC	42.77	11.92
02.058	43.55	2.21
02.087p	44.63	6.07

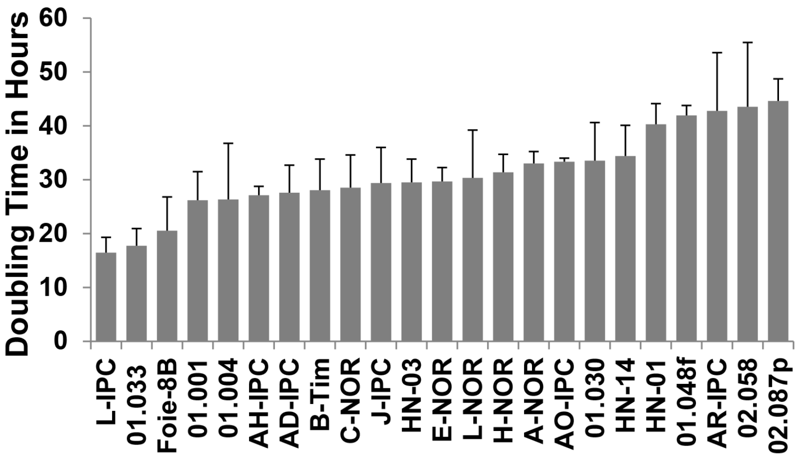

Supplementary Figure 1: Doubling Times for Primary cell Cultures (PCC). 5.000 primary cultured cells from each patient were quantified with PrestoBlue 24h after plating on a 96 well plate and after 3 days. These values were used to calculate the replication time every 24h.
